# Online awareness is associated with superior performance on assessments of functional cognition

**DOI:** 10.3389/fnagi.2024.1384812

**Published:** 2024-07-03

**Authors:** Timothy S. Marks, Gordon Muir Giles, Dorothy Farrar Edwards

**Affiliations:** ^1^Department of Occupational Therapy, University of Missouri, Columbia, MO, United States; ^2^Department of Occupational Therapy, Samuel Merritt University, Oakland, CA, United States; ^3^Departments of Kinesiology and Medicine, University of Wisconsin-Madison, Madison, WI, United States

**Keywords:** awareness, functional cognition, performance-based testing, IADL, cognition

## Abstract

**Background:**

Intact awareness facilitates an individual’s adoption of strategies to support community living skills. However, most studies have not examined awareness during ongoing complex task performance. Objective: To examine whether community-dwelling adult’s Accuracy and Strategy use on the Weekly Calendar Planning Activity 17-item version (WCPA-17), Total Cues on the Performance Assessment of Self-care Skills Checkbook Balancing and Shopping Task (PCST), and scores on the self-report Alzheimer’s Disease Cooperative Study – Activities of Daily Living Scale (ADCS-ADL) differ between groups who do and do not demonstrate awareness of performance difficulties on the WCPA-17.

**Methods:**

Using data collected as part of a larger study we performed a cross-sectional analysis of 274 community-dwelling adults aged 55 to 93 years. Two methods classified participants into groups aware or unaware of their performance. Independent sample *t*-tests examined group differences on four dependent variables: Accuracy and Strategy use on the WCPA-17, PCST Total Cues, and score on the ADCS-ADL.

**Results:**

Using one classification method, aware individuals showed superior Accuracy (*p* < 0.001), used more Strategies (*p* = 0.002), needed fewer PCST Total Cues (*p* < 0.001), and reported greater independence on the ADCS-ADL (*p* < 0.004), similar trends were observed with the other method in Accuracy (*p* < 0.001) and PCST Total Cues (*p* < 0.001) but Strategy use and ADCS-ADL differences failed to reach significance after Bonferroni correction.

**Conclusion:**

Groups categorized as aware performed better on all measures. Intact awareness is critical to performance on complex everyday activities and can be evaluated with functional cognition assessments.

## Introduction

Individuals over 55 years of age may experience changes in the ability to perform cognitively demanding everyday activities such as medication management, scheduling appointments, or managing finances (Alexander et al., 2015). Both subjective cognitive decline and subtle indicators of decrements in performance (preclinical disability), such as taking longer or having more difficulty with complex activities, may indicate a potential for the development of mild cognitive impairment in a subset of individuals (MCI; Albert et al., 2011; Petersen et al., 2014). Older adults aware of their increasing difficulty on complex tasks may limit their activities or adopt other compensatory strategies to maximize their independent functioning (Baltes and Baltes, 1990). Evidence suggests that the use of compensatory strategies amongst older adults is associated with greater levels of functional independence despite cognitive impairment (Tomaszewski Farias et al., 2018). Thus, awareness of increased difficulty when performing everyday activities is a prerequisite for developing and using compensatory strategies and there is evidence that older adults have diminished awareness of errors on both laboratory measures and in daily life (Harty et al., 2013). Older adults’ failure to recognize limitations can negatively affect performance areas critical to everyday functioning such as financial management and driving (Steward et al., 2019; Rosenfeld et al., 2022). Awareness has also been associated with older adults change in strategies to avoid potential falls (Vincenzo et al., 2022).

As a result of its central position in the maintenance of independent living skills, awareness has been studied extensively and is recognized as a complex multi-level construct with models developed by various disciplines including neuropsychology, cognitive psychology, and social psychology (Toglia and Goverover, 2022). A highly influential early model developed by Crosson et al. (1989) described hierarchical levels of awareness (intellectual, emergent, and anticipatory awareness) in individuals with brain injury. The model of awareness adopted here, the Dynamic Comprehensive Model of Awareness (DCMA; Toglia and Kirk, 2000), expands upon the work by Crosson et al. (1989) and conceptualizes awareness as having offline and online components. The DCMA model has since been used to examine awareness across different diagnostic groups receiving rehabilitation (Toglia and Goverover, 2022). According to the DCMA, offline awareness is comprised of general self-knowledge of the individual’s abilities that can be assessed via a questionnaire or interview. In contrast, online awareness is activated only during the performance of a specific task and is best assessed during or immediately following task performance (Toglia and Kirk, 2000). Online awareness relates to an individual’s ability to use self-knowledge about their own difficulties, limitations, and impairments to monitor and adjust performance while actively engaged in executing the task. Online awareness is commonly measured using performance discrepancy methods that compare client self-ratings with external criterion such as test administrator ratings or objective test scores (Toglia and Maeir, 2018). Given the significant influence of awareness on everyday function, Hartman-Maeir et al. (2009) noted that online awareness can be task specific and recommended that awareness be evaluated across contexts using performance-based assessments of functional cognition. Functional cognition assessments focus on the application of cognition to real life contexts to assess individuals who may have difficulty performing real life tasks (Wolf et al., 2019). However, comprehensive assessments that can be used to evaluate online awareness need further investigation regarding their relationship to strategy use, complex task performance, and offline awareness.

The Weekly Calendar Planning Activity (WCPA; Toglia, 2015) is a performance-based assessment of functional cognition that allows for measurements of online awareness via an after-task interview. The mid-level difficulty version of the WCPA involves scheduling a list of 17 appointments (WCPA-17) into a weekly calendar while adhering to rules, monitoring time, and resolving conflicting task demands. Administration of the WCPA-17 allows for the development of a performance profile for the test-taker that identifies various aspects of performance (e.g., Accuracy, Strategy use, etc.) relative to stratified normative values. Designed to guide strategy-based interventions, the WCPA-17 can include the examination of error type and noting of the various strategies used during performance (Toglia and Foster, 2021a). The after-task interview provides additional information on awareness, strategy use, and challenges or difficulties recognized by the test-taker. Using the WCPA, online awareness has been examined in community-dwelling and clinical populations. On a 10-item version of the WCPA (WCPA-10), community-dwelling adults with decreased online awareness were less accurate in scheduling appointments and used fewer strategies to aid WCPA performance (Arora et al., 2021). Adult patients with stroke were found to overestimate their performance and used fewer strategies during performance than healthy controls (Jaywant et al., 2022). Altered online awareness was associated with worse performance in both groups. In healthy adolescents, better awareness on the WCPA-17 was associated with better accuracy scores (Zlotnik and Toglia, 2018), and in adolescents with attention-deficit/hyperactivity disorder awareness on the WCPA-17 was significantly lower than that of controls (Fisher et al., 2022). Prior research using the WCPA has not related awareness to performance on a measure external to the WCPA itself.

In this study, we examined the relationship of online awareness to Accuracy and Strategy use on the WCPA-17, the Performance Assessment of Self-care skills Checkbook Balancing and Shopping Task (PCST; i.e., a criteria external to the WCPA-17), and a self-report measure of community living skills, the Alzheimer’s Disease Cooperative Study – Activities of Daily Living Scale (ADCS-ADL).

Two distinct but related indicators of awareness during performance on the WCPA-17 were used in this study. The first method compared the difference between the participant’s self-ratings (SR) of performance to actual performance, and the second method used the discrepancy score (DS) between the actual score and the participant’s estimate of their score. Using aware and unaware groups, we tested the following hypotheses: (1) individuals in the aware groups will have significantly higher WCPA-17 Accuracy scores than individuals in the unaware groups, (2) individuals in the aware groups will use significantly more Strategies on the WCPA-17 than individuals in the unaware groups, (3) individuals in the aware groups will have significantly fewer Total Cues on the PCST than individuals in the unaware groups, and (4) individuals in the aware groups will have significantly higher ADCS-ADL scores than individuals in the unaware groups. We further hypothesized that these group differences would be maintained after controlling for demographic variables.

## Materials and methods

### Research design

This cross-sectional study uses data gathered as part of the Menu Task validation study (Marks et al., 2020, 2021a,b; Al-Heizan et al., 2022).

### Participants and recruitment

Data collected for the primary study and examined in this study were obtained from a convenience sample of 287 community-dwelling adults recruited in Madison, Wisconsin, and the surrounding area. Inclusion criteria were age 55 years or older, living independently in the community, willingness and ability to read and write in English, and vision, hearing, and motor skills adequate for testing. The study was approved by the University of Wisconsin – Madison Institutional Review Board, and all participants provided written informed consent before participating in the study.

### Measures

#### Performance-based functional cognition measures

##### 17-item Weekly Calendar Planning Activity (WCPA-17)

The WCPA is a performance-based assessment that examines how deficits in functional cognition affect a person’s ability to perform the complex activity of entering appointments into a weekly calendar (Toglia, 2015). The 17-item version (WCPA-17) used for this study requires scheduling 17 randomly ordered appointments on a one-week calendar while minimizing errors, managing potential scheduling conflicts, and adhering to five prespecified rules. Multiple scores are derived from the WCPA, including Accuracy, Rules, Appointments Entered, Strategies, Planning time, Total time, and Efficiency. Higher scores on Accuracy, Appointments Entered, Strategies, and Rules indicate better performance. A lower Efficiency score indicates better performance. The WCPA is widely used with older adults for whom normative values have been published (Toglia, 2015). Accuracy score was used to represent the WCPA-17 because it is the primary indicator of overall task competency, and Strategy use has been hypothesized to be influenced by awareness (Toglia, 2015).

##### Classifying awareness on the WCPA-17

Two indicators of awareness during performance on the WCPA-17 were used in this study. The first method compared subjective self-ratings (SR) of performance to actual performance, and the second method used the discrepancy score (DS) between the actual score and the estimated score.

###### Actual performance compared to self-ratings of performance (SR)

Based on the method described in Arora et al. (2021), subjective self-ratings of performance were compared to actual performance. During an after-task interview, participants ranked their agreement with the following statements, “This task was easy for me,” “I used efficient methods to complete this task,” “I completed this task accurately,” and “I kept track of everything I needed to do” using a four-point Likert scale (1 = agree, 2 = somewhat agree, 3 = somewhat disagree, 4 = disagree). An average score was calculated for each individual, and participants were dichotomized into those who on average agreed with the statements (≤ 2) and those who on average disagreed (>2). We used the median Accuracy score on the WCPA-17 to further classify individuals into those with better and worse performance. The following groups were established: Aware-SR = individuals who rated the task as easy and scored at or above the median (Accuracy score ≥ 12, *n* = 108), or individuals who rated the task as difficult and scored below the median (Accuracy score < 12, *n* = 50), and Unaware-SR = individuals who rated the task as easy but scored below the median (*n* = 86). Consistent with the procedure described by Arora et al. (2021), participants who rated the task as difficult and scored at or above the median Accuracy score of the sample were placed in the Aware-SR group (*n* = 30).

###### Actual score compared to estimated score: discrepancy score (DS)

The difference between the actual number of accurate appointments and the participant’s estimation of the number of accurate appointments during an after-task interview resulted in a discrepancy score. Perfect agreement between the participant’s estimate and their actual score produced a score of zero. By subtracting the actual score from the estimated score, a score of less than zero indicated that the participant over-estimated their actual performance. A score of greater than zero indicated an underestimation of their actual performance. Following Arora et al. (2021), participants were classified as Aware-DS or Unaware-DS based on the median estimation discrepancy of the sample (Mdn = −4). Participants who underestimated their performance relative to the median discrepancy score were included in the Aware-DS group (Arora et al., 2021).

#### Performance Assessment of Self-Care Skills (PASS) Checkbook Balancing and Shopping Task (PCST)

The PASS (Rogers et al., 2016) includes 26 items that measure activities of daily living (ADL) and instrumental activities of daily living (IADL) skills intended to assist clinicians in planning interventions, 14 of which are described as having a cognitive emphasis (C-IADL). The PASS Checkbook Balancing and Shopping tasks (PCST) were used for this study. The Checkbook Balancing task requires participants to enter checks into a checkbook ledger and then to balance the checking account. The Shopping task requires participants to use money and coupons to select and purchase food items from a shopping list (Rogers et al., 2016). The PCST has been established as a measure of preclinical disability and is as sensitive as the combined 14-IADL tasks in discriminating between individuals with MCI and healthy older adults (Rodakowski et al., 2014). PCST scores are based on the combined number of cues required for independence and adequacy on each task (i.e., Total Cues), with lower numbers indicating better performance (Rogers et al., 2016).

### Self-report ADL/IADL measure

#### Alzheimer’s Disease Cooperative Study – Activities of Daily Living Inventory Scale (ADCS-ADL)

The ADCS-ADL consists of 23 informant or self-report items designed to evaluate ADL and IADL performance in individuals enrolled in clinical trials (Galasko et al., 1997). The ADCS-ADL has shown good test–retest reliability, correlates with cognitive screening measures in individuals with MCI and Alzheimer’s disease (AD) and is sensitive to disease progression (Galasko et al., 1997; Doraiswamy et al., 2014; Cintra et al., 2017). Informant and proxy ratings have been found to be highly correlated on the ADCS-ADL (Howland et al., 2017). Higher scores indicate greater independence.

#### Study procedures

Testing occurred per participant convenience at either community settings or at the Occupational Therapy Department at the University of Wisconsin – Madison. Demographic information including age, sex, race, education (in years), and the number of self-reported chronic health conditions were collected from participants. Testing was completed by trained occupational therapy graduate students in quiet, office-like environments. All data were collected in one study visit. Participants were allowed to take breaks if needed during testing. Participants received remuneration of $25 in cash.

#### Analysis

Descriptive statistics were computed for continuous variables and frequency distributions for categorical variables. All variables were inspected for normality to guide analysis. Total sample mean values were used to replace three values of isolated, missing demographic information. The number of chronic health conditions and the PCST Total Cues score were log transformed to reduce right skew and the ADCS-ADL scores were reflected and then log transformed to reduce left skew. The transformations resulted in normal distributions for the variables.

Two independent methods described above were used to divide the sample into “aware” and “unaware” groups according to the WCPA-17 categorizations. The association between the awareness categorization methods (i.e., Aware-SR or Unaware-SR, and Aware-DS or Unaware-DS) was examined using Spearman’s correlation coefficient. Pearson correlation coefficients were used to examine the linear relationships between Strategies used and performance (Accuracy on the WCPA-17, PCST Total Cues, and ADCS-ADL) for the overall sample and for each awareness group.

Independent groups *t*-tests and chi-squared analyses were used to examine differences in demographic characteristics between the aware groups and the unaware groups.

Accuracy scores and number of Strategies used on the WCPA-17, PCST Total Cues, and ADCS-ADL scores were compared between the aware and unaware groups using independent groups *t*-tests. To reduce the risk of Type 1 error, Bonferroni corrections were used for multiple comparisons with a *p*-value set at 0.006 to indicate significance (0.05/8 = 0.006). Estimates of effect sizes were quantified via Cohen’s *d* value to determine the magnitude of significant group differences and were interpreted as: 0.2 = small, 0.5 = medium, and 0.8 = large (Cohen, 1988). Demographic variables were examined to assess whether a linear relationship existed with scores on the study measures. Where a correlation of 0.30 or higher (Portney, 2020) was present the influence of such demographic variables on group comparisons were examined. Accuracy and Strategy use on the WCPA-17, PCST Total Cues, and ADCS-ADL scores were compared between the aware and unaware groups using one-way analysis of covariance (ANCOVA) models to control for the potential demographic effects. SPSS version 27 was used for all analyses (IBM Corp, 2020).

## Results

Thirteen individuals were dropped from the analysis due to not completing the PCST or the WCPA-17 leaving a final sample size of 274 participants. The demographic characteristics of the sample are presented in Table 1. Participants were predominantly female (73.72%) and White (81.02%). The mean age of the sample was 69.59 (SD = 8.12) years, with an average education of 15.58 (SD = 3.27) years. The total sample had mean Accuracy scores of 10.66 (SD = 4.35) on the WCPA-17 and used an average of 5.05 (SD = 2.01) Strategies on the WCPA-17. The mean Total Cues on the PCST was 9.45 (SD = 9.05). The mean score on the ADCS-ADL was 75.04 (SD = 4.04).

**Table 1:** Demographic characteristics and scores on study measures.

	Full sample *N* = 274
Variable	*M (SD)*
Age	69.59 (8.12)
Chronic health conditions	1.04 (1.19)
Education	15.58 (3.27)
	*n* (%)
Sex	
Female	202 (73.72)
Male	72 (26.28)
Race	
White	222 (81.02)
Black/African American	36 (13.14)
Other	16 (5.84)
	*M (SD)*
Measures	
WCPA-17 accuracy	10.66 (4.35)
WCPA-17 strategies	5.05 (2.01)
PCST total cues	9.45 (9.05)
ADCS-ADL	75.04 (4.04)

WCPA-17, Weekly Calendar Planning Activity 17-item; PCST, Performance Assessment of Self-care Skills Checkbook Balancing and Shopping Task; ADCS-ADL, Alzheimer’s Disease Cooperative Study Activities of Daily Living Scale.

A total of 191 individuals were classified as Aware-SR and 83 individuals as Unaware-SR, and a total of 164 individuals were classified as Aware-DS and 110 individuals as Unaware-DS. The two methods agreed on the identification of 155 individuals as aware, and 74 individuals as unaware. The SR method identified 3% individuals (n = 9) as unaware that were not so identified by the DS method, and the DS method identified 13% individuals (*n* = 36) as unaware that were not so identified by the SR method. Overall, there was 84% agreement and a significant correlation between the two categorizations of awareness (*r*_s_ = 0.659, *p* < 0.001). Correlations for the total sample between Strategies used on the WCPA-17 and performance on the WCPA-17 Accuracy were significant and low to fair (*r* = 0.36, *p* < 0.01). Table 2 presents the correlational results. Correlations between Strategies used on the WCPA-17 and the PCST Total Cues (*r* = −0.24, *p* < 0.01) and the ADCS-ADL (*r* = −0.18, p < 0.01) were also significant but showed little to no relationship.

**Table 2:** Correlation between strategies used and performance on the WCPA-17, PCST Total Cues, and ADCS-ADL.

Variable	WCPA-17 accuracy	PCST total cues	ADCS-ADL
Strategies used			
Total sample (N = 274)	0.36*	−0.24*	−0.18*
Aware-SR group (n = 191)	0.38*	−0.23*	−0.18
Unaware-SR group (n = 83)	0.12	−0.14	−0.08
Aware-DS group (n = 164)	0.39*	−0.22*	−0.16
Unaware-DS group (n = 110)	0.27*	−0.19	−0.15

WCPA-17, Weekly Calendar Planning Activity 17-item; PCST, Performance Assessment of Self-care Skills Checkbook Balancing and Shopping Task; ADCS-ADL, Alzheimer’s Disease Cooperative Study Activities of Daily Living Scale.*p < 0.01.

Correlations for the aware groups between Strategies used on the WCPA-17 and performance on the WCPA-17 Accuracy and the PCST using both methods of categorization were higher for the aware groups than for the unaware groups, but the strength of the relationships were at best moderate (Portney, 2020).

### Self-rating of performance

Individuals in the Unaware-SR group had significantly fewer years of education (*p* = 0.001) and more self-reported chronic health conditions (*p* = 0.011) than those in the Aware-SR group. There was a significant association between sex and awareness categorization (*p* = 0.032), no significant difference between race and awareness categorization (*p* = 0.474) and no significant difference in age existed between the Aware-SR and the Unaware-SR groups (*p* = 0.304; see Table 3).

**Table 3:** Comparison between aware and unaware groups using two methods of categorization on demographics and assessment scores.

	Self-rating of Performance				Discrepancy Score			
	Aware-SR (*n* = 191)	Unaware-SR (*n* = 83)			Aware-DS (*n* = 164)	Unaware-DS (*n* = 110)		
	*M (SD)*	*M (SD)*	*t_(1,272)_*; *p* =	*d*	*M (SD)*	*M (SD)*	*t_(1,272)_*; *p* =	*d*
Demographics								
Age	69.23 (7.64)	70.41 (9.12)	−1.03; 0.304	0.145	68.77 (7.18)	70.80 (9.25)	−1.94; 0.054	0.251
Chronic health conditions	0.92 (1.09)	1.31 (1.36)	−2.57; 0.011*	0.338	0.93 (1.12)	1.21 (1.27)	−2.19; 0.035*	0.261
Education	16.02 (3.36)	14.57 (2.82)	3.43; < 0.001*	0.451	16.18 (3.31)	14.67 (3.01)	3.84; < 0.001*	0.473
	*n* (%)	*n* (%)			*n* (%)	*n* (%)		
Sex								
Female	148 (77.49)	54 (65.06)	χ^2^ = 4.61; 0.032*		122 (74.39)	80 (72.73)	χ^2^ = 0.09; 0.759	
Male	43 (22.51)	29 (34.94)			42 (25.61)	30 (27.27)		
Race								
Black	22 (11.52)	14 (16.87)	χ^2^ = 1.49; 0.474		15 (9.15)	21 (19.09)	χ^2^ = 5.99; 0.050	
White	158 (82.72)	64 (77.11)			140 (85.36)	82 (74.55)		
Other	11 (5.76)	5 (6.02)			9 (5.49)	7 (6.36)		
Performance-based measures								
WCPA-17 accuracy	12.07 (3.94)	7.41 (3.40)	9.37; < 0.001**	1.231	12.79 (3.44)	7.49 (3.56)	12.23; < 0.001**	1.517
WCPA-17 strategies	5.29 (2.00)	4.49 (1.93)	3.06; 0.002**	0.402	5.29 (2.00)	4.68 (1.96)	2.50; 0.013*	0.307
PCST total cues	8.10 (8.59)	12.55 (9.38)	−4.38; < 0.001**	0.575	7.20 (6.47)	12.81 (11.12)	−4.88; < 0.001**	0.601
Self-report measure								
ADCS-ADL	75.48 (3.07)	74.02 (5.56)	−2.92; 0.004**	0.384	75.52 (2.92)	74.32 (5.20)	−2.38; 0.018*	0.294

WCPA-17, Weekly Calendar Planning Activity 17-item; PCST, Performance Assessment of Self-care Skills Checkbook Balancing and Shopping Task; ADCS-ADL, Alzheimer’s Disease Cooperative Study Activities of Daily Living Scale; d = Cohen’s d, 0.1 = small, 0.5 = medium, 0.8 = large (Cohen, 1988).^*^p < 0.05.^**^Bonferroni correction, *p* ≤ 0.006.

There were statistically significant differences on Accuracy (*p* < 0.001) and Strategies used (*p* = 0.002) on the WCPA-17, PCST Total Cues (*p* < 0.001), and self-rating on the ADCS-ADL (*p* = 0.004) between the Aware-SR and the Unaware-SR groups. Effect sizes for the group differences ranged from small to large. Table 3 presents the main results of the group comparisons. Of the demographic variables, only education and WCPA-17 Accuracy and education and PCST Total Cues were correlated at 0.30 or above. Using an ANCOVA model to examine group differences while adjusting for education, Accuracy scores (*F* (1, 271) = 72.56, *p* < 0.001) and PCST Total Cues (*F* (1, 271) = 12.30, *p* < 0.001) remained significantly different between the Aware-SR and the Unaware-SR groups.

### Discrepancy score

Individuals in the Unaware-DS group had significantly fewer years of education (*p* = 0.001) and more self-reported chronic health conditions (*p* = 0.035) than those categorized as Aware-DS, though no significant difference in age (*p* = 0.054), sex (*p* = 0.759), and race (*p* = 0.050) existed between the Aware-DS and the Unaware-DS groups, though the association between race awareness classification approached significance (see Table 3).

There were statistically significant differences in Accuracy (*p* < 0.001) on the WCPA-17 and PCST Total Cues (*p* < 0.001) between the Aware-DS and the Unaware-DS groups. More Strategies were used on the WCPA-17 (*p* = 0.013) and more independence was reported on the ADCS-ADL (*p* = 0.018) by the Aware-DS compared to the Unaware-DS groups; however, these differences were no longer significant after Bonferroni correction. Effect sizes for the group differences ranged from small to large. Table 3 presents the main results of the group comparisons. Of the demographic variables, only education and WCPA Accuracy and education and PCST Total Cues were correlated at 0.30 or above. Using an ANCOVA model to examine group differences while adjusting for education, Accuracy (*F* (1, 271) = 129.94, *p* < 0.001) and PCST Total Cues (*F* (1, 271) = 15.31, *p* < 0.001) remained significantly different between the Aware-DS and the Unaware-DS groups.

## Discussion

The primary purpose of this study was to determine if Accuracy and Strategy scores on the WCPA-17, PCST Total Cues, and ADCS-ADL scores differed by online awareness category using two distinct but related methods to categorize awareness. As hypothesized, the groups categorized as aware had significantly better Accuracy on the WCPA-17 compared to the unaware groups. Similarly, the aware groups used more Strategies to complete the WCPA-17; however, the differences in Strategies was no longer significant after Bonferroni correction for the Aware-DS categorization. Considered together the aware groups required significantly fewer Total Cues to complete the PCST. Scores on the ADCS-ADL were higher in the aware groups than in the unaware groups using both methods of awareness categorization, but for the Awareness-DS categorization the ADCS-ADL scores were no longer significantly higher following Bonferroni correction. Overall, these findings using distinct methods of categorizing online awareness on the WCPA-17 yielded similar results, indicating that individuals with diminished online awareness are more likely to score lower on functional cognition assessments and on a self-report measure of complex everyday activities. The findings are convergent however the DS method captures almost all the individuals identified as unaware in the SR method and categorizes more individuals as unaware and may therefore be considered more sensitive than the SR method.

Using the WCPA-10, Arora et al. (2021) found that awareness was significantly correlated with Accuracy and Strategy use in community-dwelling adults. Our study extends these findings by using the more complex WCPA-17, use of a separate functional cognition measure (PCST), and use of a self-report functional independence measure (ADCS-ADL). To examine the influence of demographic factors, we ran correlations between age, education, chronic health conditions, and our outcome variables. Given the moderate correlation with education, its influence was assessed using ANCOVA analyses. All significant differences between the awareness groups remained after controlling for education.

The effect sizes for group differences in Accuracy on the WCPA-17 and Total Cues on the PCST in our study are indicative of the moderate to strong relationships between online awareness and performance of complex daily living skills examined by the performance-based measures. Accuracy on the WCPA-17 is an indication of the overall ability to perform the task while managing associated unexpected difficulties and challenges. The PCST measures functional cognition but does so with a distinct content, cueing, and scoring system. Therefore, these results have practical implications that extend past the WCPA-17, as the ability to complete complex everyday tasks such as those assessed with the PCST safely and without errors is important for community independence.

Awareness influences performance of everyday activities (Shaked et al., 2019) and is related to the need to select and use strategies effectively to enhance performance (Toglia et al., 2012). Individuals with and without cognitive impairment who use a greater number of strategies to complete complex everyday activities such as managing appointments, shopping, cooking, managing finances, transportation, and managing medications report greater independence during the performance of these activities (Tomaszewski Farias et al., 2018). As such, interventions have been advanced in the rehabilitation literature that promote the learning and understanding of strategies to enhance performance during everyday activities (Polatajko and Mandich, 2004; Toglia and Foster, 2021b). Although individuals use strategies to manage many aspects of daily life, strategies are particularly important during the performance of complex everyday activities because the activities themselves have high cognitive demands and can be further complicated by contextual, personal, and psychological variables (Toglia and Kirk, 2000). Using strategies to complete challenging or novel everyday activities aids in adapting performance to these varying demands and can help with efficiency, speed, accuracy, and consistency of task performance (Toglia et al., 2012). In this study, online awareness on the WCPA-17 was associated with the use of significantly more strategies to support the accurate entry of appointments into the weekly calendar. In the correlational analyses, the strength of the relationship between Accuracy and Strategies – a measure of the number of strategies used but not the efficiency of their use—were higher for both the overall sample and the aware group. These findings are similar to associations observed in a recent normative study (Arora et al., 2021). The limited association between Accuracy and the number of Strategies used points to other potential unknown metacognitive factors in relation to strategy use that may be more relevant to performance such as how the individual uses the strategies they attempt. There was little to no association between Accuracy and Strategy use in the unaware groups. Individuals who were unaware of their performance may not have been able to use selected strategies appropriately or make changes in strategy use dependent on ongoing performance errors. This potential difference in strategy use highlights the importance of capturing more details of performance such as the quality and effectiveness of strategy use, information which could then be used for intervention planning. Individuals with altered awareness in this study could benefit from interventions that aim to increase awareness and metacognitive skills, including the ability to identify and use effective strategies to support performance.

Individuals unaware of their performance on the WCPA-17 scored significantly worse on a separate measure of functional cognition, the PCST. The moderate effect size of this difference, in contrast to the large effect size seen with Accuracy on the WCPA-17, points to the potential influence of task characteristics on online awareness and hence the importance of evaluating awareness specific to the task itself. We evaluated online awareness, which involves judgments in relation to a specific situation (in this case, the WCPA-17) and which may be highly dependent on task characteristics such as familiarity and complexity (Toglia and Goverover, 2022). The finding that performance on the PCST was associated with online awareness described by the WCPA-17 suggests that individuals who are unaware of ongoing task performance may be at risk for poor performance during other complex everyday tasks that present similar cognitive and functional challenges, though this finding needs further exploration as awareness can also be task specific (Goverover et al., 2007; Sansonetti et al., 2024). Poor online awareness provides clinicians with an additional datapoint over and above performance Accuracy in making judgements about an individual’s functional independence and need for support. Of note, the aware groups reported greater independence on the ADCS-ADL. Although consistent with findings on Accuracy and Strategies used on the WCPA-17 and PCST Total Cues, we have no objective external measure to validate this finding (i.e., comparison to informant report, direct observation of ADL/IADL). It may be that this finding is accurate and the groups that were unaware of their online ability during the WCPA-17 had maintained intact offline awareness and were able to accurately report their independence skills, however, this result should be interpreted with caution. Inconsistencies have been found between offline awareness and online awareness abilities across diagnostic populations, supporting the idea that awareness is not a unitary construct. Assessing differing aspects of awareness could provide distinctive value (Toglia and Goverover, 2022) and future studies could compare the associations between offline and online awareness of function in community-dwelling adults. Future studies should examine the relationship between performance-based measures that include evaluation of awareness in older adults such as the WCPA-17 and other areas of functioning where awareness is critical to performance such as driving safety and financial decision-making (Paire-Ficout et al., 2021; Yu et al., 2022; Mazzonna and Peracchi, 2024).

As expected, the groups categorized as aware performed better on all functional cognition performance scores than the groups categorized as unaware. Online awareness is critical to performing complex everyday activities and can be evaluated with functional cognition assessments. The WCPA-17 can be used to evaluate awareness in the context of complex everyday performance and may be useful for assessment and intervention planning. The current work does have limitations. Most participants in this study were highly educated, White, and from one geographic region of the United States. These factors limit the generalizability of the findings. The adequacy of Strategies used to complete the WCPA-17 were not formally evaluated, and future studies should seek to capture more descriptive information regarding strategy use during administration of the WCPA. We used two methods of characterizing awareness in this study to provide for greater confidence in our findings and to provide clinicians with alternate methods of assessing awareness with clients with whom they are using the WCPA-17, but various indicators of online awareness exist that could also be examined and may more clearly describe the relationship between online awareness and performance. Future studies should collect participant information on affective symptoms and performance on neuropsychological tests of cognition. Additional studies are needed to replicate these results in additional populations and to further examine the relationships between awareness and functional performance.

## Data availability statement

The raw data supporting the conclusions of this article will be made available by the authors, without undue reservation.

## Ethics statement

The studies involving humans were approved by Institutional Review Board of the University of Wisconsin - Madison. The studies were conducted in accordance with the local legislation and institutional requirements. The participants provided their written informed consent to participate in this study.

## Author contributions

TM: Conceptualization, Data curation, Investigation, Methodology, Supervision, Writing – original draft, Writing – review & editing. GG: Conceptualization, Data curation, Investigation, Methodology, Writing – original draft, Writing – review & editing. DE: Conceptualization, Data curation, Investigation, Methodology, Project administration, Supervision, Writing – original draft, Writing – review & editing.
